# Bile Reflux is a Common Finding in the Gastric Pouch After One Anastomosis Gastric Bypass

**DOI:** 10.1007/s11695-019-04353-x

**Published:** 2019-12-18

**Authors:** Tuure Saarinen, Kirsi H. Pietiläinen, Antti Loimaala, Toni Ihalainen, Henna Sammalkorpi, Anne Penttilä, Anne Juuti

**Affiliations:** 1 Department of Gastrointestinal Surgery, Helsinki University Hospital, Abdominal Center, Helsinki, Finland; 2 HUS Jorvi Hospital, Turuntie 150, 02740 Espoo, Finland; 3 Department of Gastrointestinal Surgery, Helsinki University Hospital, Abdominal Center, Haartmaninkatu 4, 00029 HUS Helsinki, Finland; 4 Research Program for Clinical and Molecular Metabolism, Faculty of Medicine, University of Helsinki, Haartmaninkatu 4, 00029 HUS Helsinki, Finland; 5 Department of Endocrinology, Helsinki University Hospital, Abdominal Center, Haartmaninkatu 4, 00029 HUS Helsinki, Finland; 6 HUS Medical Imaging Center, Clinical Physiology and Nuclear Medicine, University of Helsinki and Helsinki University Hospital, Haartmaninkatu 4, 00029 HUS Helsinki, Finland

**Keywords:** One anastomosis gastric bypass, Bile reflux, Bariatric surgery, Upper gastrointestinal endoscopy, Scintigraphy

## Abstract

**Introduction:**

Data on postoperative bile reflux after one anastomosis gastric bypass (OAGB) is lacking. Bile reflux scintigraphy (BRS) has been shown to be a reliable non-invasive tool to assess bile reflux after OAGB. We set out to study bile reflux after OAGB with BRS and endoscopy in a prospective series (*RYSA* Trial).

**Methods:**

Forty patients (29 women) underwent OAGB between November 2016 and December 2018. Symptoms were reported and upper gastrointestinal endoscopy (UGE) was done preoperatively. Six months after OAGB, bile reflux was assessed in UGE findings and as tracer activity found in gastric tube and esophagus in BRS (follow-up rate 95%).

**Results:**

Twenty-six patients (68.4%) had no bile reflux in BRS. Twelve patients (31.6%) had bile reflux in the gastric pouch in BRS and one of them (2.6%) had bile reflux also in the esophagus 6 months postoperatively. Mean bile reflux activity in the gastric pouch was 5.2% (1–21%) of total activity. De novo findings suggestive of bile reflux (esophagitis, stomal ulcer, foveolar inflammation of gastric pouch) were found for 15 patients (39.5%) in postoperative UGE. BRS and UGE findings were significantly associated (*P* = 0.022). Eight patients experienced de novo reflux symptoms at 6 months, that were significantly associated with BRS and de novo UGE findings postoperatively (*P* = 0.033 and 0.0005, respectively).

**Conclusion:**

Postoperative bile reflux in the gastric pouch after OAGB is a common finding in scintigraphy and endoscopy. The long-term effects of bile exposure will be analyzed in future reports after a longer follow-up.

**Trial registration:**

Clinical Trials Identifier NCT02882685

## Introduction

The bariatric surgery community has seen a rise of one anastomosis gastric bypass (OAGB) during the last years. In 2016, OAGB was the third most common bariatric operation constituting 4.8% of all primary operations after sleeve gastrectomy (53.6%) and Roux-en-Y gastric bypass (RYGB) (30.1%) [1]. According to the 5th IFSO registry report of 2019, OAGB constituted 3.7% of all primary bariatric procedures. Many reports have shown excellent weight loss results after OAGB, but throughout OAGB’s over 20-year history, there has been a lot of debate on the safety of the procedure [2, 3]. To date, two prospective randomized controlled trials comparing OAGB and RYGB have been published [4, 5]. In a newly published review of these two trials, Lee et al. concluded that the controversy of bile reflux requires elucidation after longer follow-up [6]. Bile reflux has been feared to even cause cancer in the esophagus or gastric pouch, and it is the main reason why many surgeons do not perform OAGB procedures according to a recent survey [7]. Evidence of carcinogenic effect of bile reflux after OAGB is lacking, but postoperative de novo reflux symptoms after OAGB are quite common [3].

Our group published a pilot series on studying bile reflux with a scintigraphic method in 2017 [8]. We found that bile reflux can be assessed accurately by using a non-invasive scintigraphic method. This was also confirmed in a review in 2018 [9]. Here, we set out to prospectively study bile reflux with a scintigraphic method (bile reflux scintigraphy, BRS) and endoscopies in a prospective series as a part of a randomized controlled trial comparing OAGB and RYGB (*RYSA* trial, clinicaltrials.org identifier NCT02882685).

## Methods

*RYSA* trial is a randomized controlled open-label trial of two academic centers comparing OAGB and RYGB. Entire *RYSA* trial design and further end-points will be described in detail in future reports. Here, we describe one part of the trial that addresses the issue of bile reflux after OAGB. Forty patients were included after randomization. All patients underwent OAGB between November 2016 and December 2018 in an academic bariatric surgery center. The authors (TS, AJ, HS, and AP) interviewed all patients preoperatively and also 6 months after OAGB). Body mass index (BMI, kg/m^2^, calculated as weight/height × height), comorbidities, and gastrointestinal symptoms were recorded prior to OAGB and 6 months after.

### Surgical Technique

OAGB was performed by the authors (AJ, HS, and AP) under general anesthesia via a standard 4-port laparoscopy and a subxiphoidal liver retractor. An approximately 15-cm long tubular gastric pouch was created along a 38-Fr bougie. Biliopancreatic limb was measured at 210 cm using a marked dissector and an antecolic anastomosis was done side-to-side with a 45-mm stapler. The remaining defect was hand-sewn with a braided absorbable 2-0 running suture including lateral antireflux stiches between gastric pouch and afferent loop of jejunum. Mesenteric defect was not closed. The common limb length was not measured.

### Scintigraphy

**Fig. 1:**
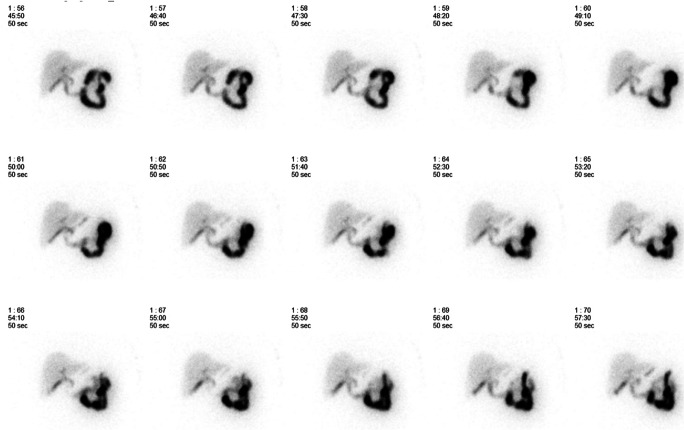
Dynamic gamma camera images of bile reflux scintigraphy. Images of a representative patient show bile tracer in the gastric pouch beginning at 51 min after intravenous administration of bile tracer (^99m^Tc-mebrofenin)

**Fig. 2:**
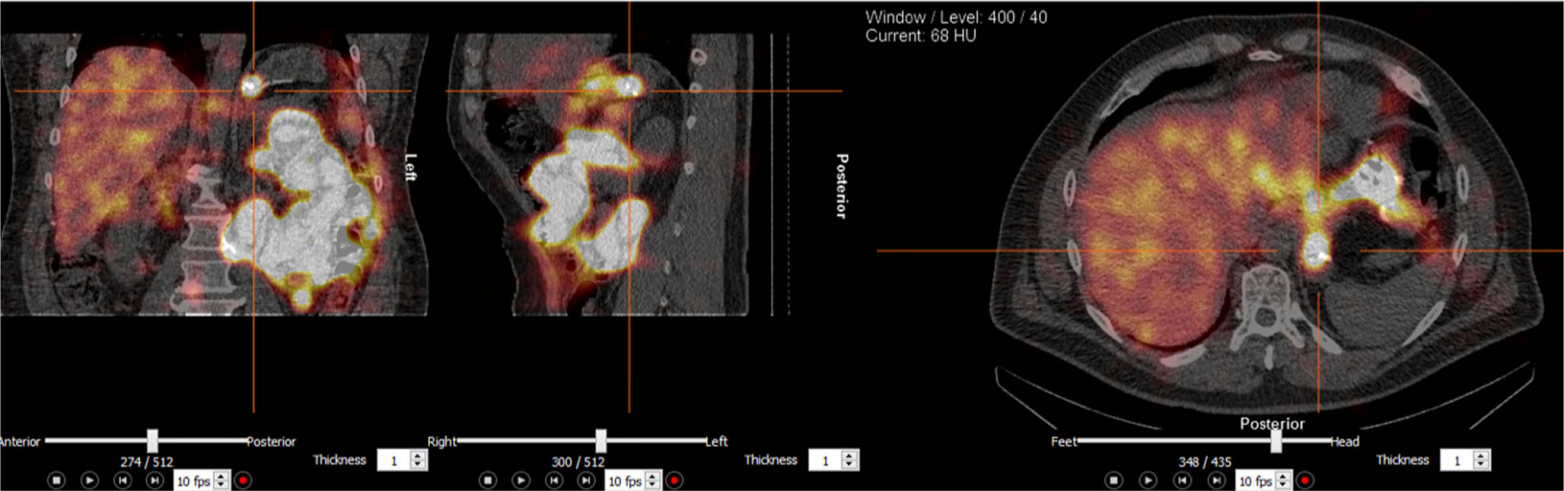
SPECT–CT scan at the end of bile reflux scintigraphy of a representative patient. Bile tracer activity in the gastric pouch and esophagus are shown

Bile reflux scintigraphy (BRS) was done at 6 months with a method that was described in detail previously [8]. In short, after 12 h of fasting, an intravenous bile tracer (^99m^Tc-mebrofenin) was administered, and a 60-min dynamic scan with a gamma camera immediately followed by a 30-min SPECT–CT scan was acquired. Using the images of the dynamic series, the beginning and the end of bile reflux activity in the gastric pouch and esophagus were recorded and the amount of bile reflux was calculated as a ratio of tracer induced maximum count rate per pixel measured in the gastric pouch or esophagus compared to the maximum count rate per pixel induced by the tracer in the whole liver. All scintigraphies were quantified by a physicist (TI) and analyzed by a nuclear medicine physician (AL). Examples of gamma camera images and SPECT–CT scan are shown in Fig. 1 and Fig. 2, respectively.

### Endoscopy

Upper gastrointestinal endoscopy (UGE) was done preoperatively and at 6 months. UGE was done without sedation using a flexible endoscope (Olympus Q190, Tokyo, Japan). Biopsies and photographs were obtained from the jejunum, anastomosis, gastric pouch, gastro-esophageal junction, and esophagus. All endoscopies were done by the authors (TS, AJ, HS, and AP).

### Ethical Approval

The trial was reviewed by the operative ethical committee of our institution and approved by scientific boards of both academic centers participating in the study. The study was carried out according to the Helsinki Declaration and informed consent was obtained for all patients.

### Statistical Analysis

All variables are expressed as mean (min–max) with percentages where applicable. The Fischer’s exact test was used to analyze dependence of categorical variables in contingency tables. Associations between categorical and continuous variables with BRS and UGE findings were analyzed with the binary logistic regression and one-sample *T* test, respectively. *P* value less than 0.05 was considered statistically significant.

## Results

Forty patients were included in this study after randomization into the OAGB group. Patient demographics are given in Table 1.

**Table 1:** Patient demographics

		Preop	6 mo
Number of patients	40		
Women	29 (72.5%)		
Age, y	44.4 (28.4–58.6)		
BMI, kg/m2		45.2 (35.4–62.0)	35.2 (26.3–49.6)
Comorbidities			
DM		14 (35%)	6 (15%)
Duration, y		6.7 (0.5–20.0)	
Oral medication only		10 (25%)	5 (12.5%)
Insulin		4 (10%)	1 (2.5%)
HTA		22 (55%)	16 (40%)
Dyslipidemia		10 (25%)	9 (22.5%)
OSAS		18 (45%)	9 (22.5%)
Arthrosis symptoms		19 (47.5%)	12 (32.5%)
Symptoms			
Reflux		6 (15%)	9 (22.5%)
Eating problems		0	4 (10%)

*BMI*, body mass index; *Preop*, prior to bariatric surgery; *6 mo*, 6 months after bariatric surgery; *y*, years; *kg*, kilograms; *m*, meters; DM, type 2 diabetes; *HTA*, hypertension; *OSAS*, obstructive sleep apnea

**Fig. 3:**
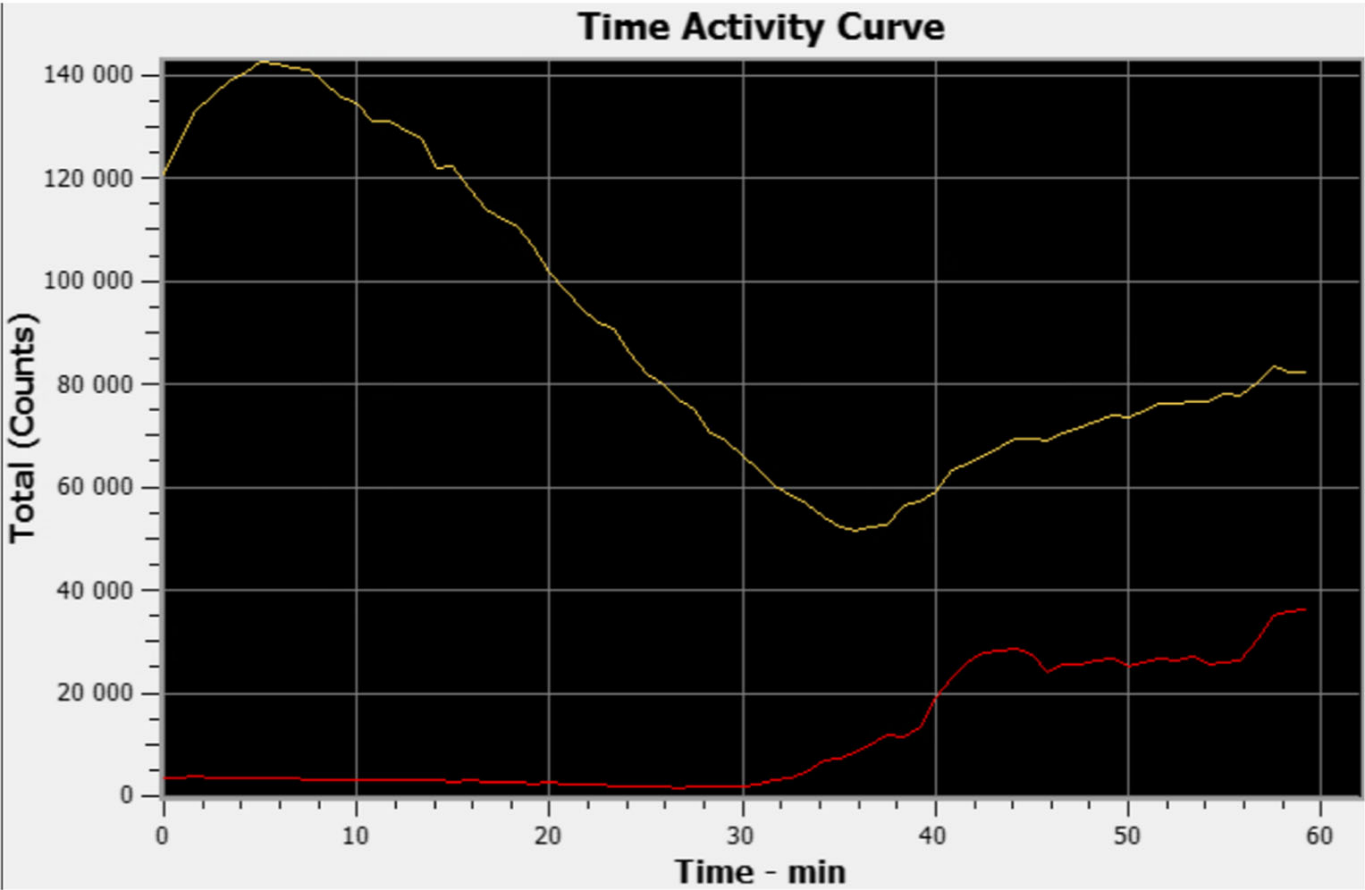
Time activity curve of the dynamic series of a bile reflux scintigraphy. Bile tracer activity in the liver (yellow line) and in the gastric pouch (red line) of one representative patient is shown. A subsequent rise in the tracer activity is seen in the gastric tube and in the liver. This is most likely due to reuptake of bile acids through enterohepatic circulation

Thirty-eight patients (95%) underwent BRS at 6 months. For 26 patients (68.4%), BRS did not show any sign of bile reflux into the gastric pouch or esophagus. Twelve patients (31.6%) had a positive BRS, 11 (28.9%) had bile reflux activity only in the gastric pouch, and one patient (2.6%) had bile reflux activity also in the esophagus at the end of the SPECT–CT scan (Fig. 2). Bile reflux activity began in the gastric pouch at 36.2 min (16–54). The highest activity during the dynamic series of BRS was at 50.2 min (38–60). Bile reflux activity did not completely stop by the end of the 90-min scan in any of the positive scintigraphies. Mean activity was 5.2% (1–21%) of total activity. For one patient, the amount of bile reflux activity could not be calculated. Figure 3 illustrates the amount of bile tracer activity during the dynamic scan in the gastric pouch and in the liver of one representative patient.

UGE was done for all 40 patients preoperatively and for 38 patients (95%) at 6 months. UGE findings are summarized in Table 2. Four patients (10.5%) had an anastomotic ulcer and two more patients had an erosive inflammation at the anastomosis. Five patients (13.2%) had de novo histologic inflammation or foveolar hyperplasia in the gastric pouch. However, these five endoscopies were considered macroscopically normal. Nine patients (23.7%) had an abnormal finding in the cardia and six patients (15.8%) had de novo esophagitis. Of these, only one esophagitis was macroscopically evident (Los Angeles grade A esophagitis). Altogether, 15 patients (39.5%) had a new abnormal postoperative finding in UGE.

**Table 2:** Histologic findings in upper gastrointestinal endoscopies preoperatively and 6 months after one anastomosis gastric bypass

UGE finding	Preop	6 mo	De novo findings at 6mo
Number of patients	40	38 (95%)	
Normal	24 (60%)	15 (39.5%)	
Anastomosis			
Inflammation		7 (18.4%)	7 (18.4%)
Ulcer		4 (10.5%)	4 (10.5%)
Foveolar hyperplasia		1 (2.6%)	1 (2.6%)
Stricture		1 (2.6%)	1 (2.6%)
Stomach/gastric pouch			
Gastritis levis	8 (20%)		
Foveolar hyperplasia or inflammation	0	8 (21.1%)	5 (13.2%)
Benign polyp	2 (5%)	0	0
*HP* positive gastritis	3 (7.5%)	0	0
Gastro-esophageal junction			
Inflammation	4 (10%)	6 (15%)	6 (15.8%)
Gastric metaplasia	1 (2.5%)	3 (7.5%)	3 (7.9%)
Intestinal metaplasia	1 (2.5%)	0	0
Esophagus			
Esophagitis	5 (12.5%)	9 (22.5%)	6 (15.8%)

UGE, upper gastrointestinal endoscopy; *Preop*, prior to bariatric surgery; *6 mo*, 6 months after bariatric surgery; *HP*, *Helicobacter pylori*

Eight patients (21.1%) had a positive BRS and a new abnormal finding in postoperative UGE, whereas 19 patients (50%) had neither bile reflux in BRS nor any new findings in postoperative UGE (*P* = 0.022).

Nine patients (22.5%) experienced reflux symptoms at 6 months. Five of them had similar symptoms preoperatively. Four patients (10%) experienced eating difficulties at 6 months. Altogether, eight patients (20%) experienced de novo symptoms at 6 months. Six of these patients had histologic esophagitis in UGE and one patient had anastomotic stricture requiring endoscopic balloon dilation. Five patients with de novo symptoms had bile reflux activity in the gastric pouch in BRS. For two patients, BRS was negative. One of the five symptomatic patients refused to have postoperative BRS and UGE. Associations between new postoperative symptoms and BRS findings as well as new abnormal UGE findings were statistically significant (*P* = 0.022 and *P* = 0.0005, respectively). No other tested variable was significantly associated with BRS or UGE findings.

## Discussion

Our current study is the first prospective study on bile reflux after OAGB using both endoscopies and scintigraphic evaluations. Our study shows that 31.6% of patients had bile reflux in the gastric pouch in BRS, and one of these patients even had bile reflux activity in the esophagus. In our previous pilot study, we found bile reflux in BRS in 55.6% of patients, but no bile reflux in the esophagus [8]. In the current study, we found new abnormal findings in postoperative UGE of 39.5% of patients. Most of these UGE findings were not evident macroscopically. Histologic findings of foveolar inflammation of the anastomosis and gastric pouch as well as gastric metaplasia and esophagitis are not specific findings of bile reflux, but may suggest effect of bile exposure in the gastric pouch and esophagus. These findings may also be at least in part due to gastro-esophageal reflux. It is possible that these findings will develop into macroscopic lesions after several years, and therefore a routine endoscopic follow-up is mandatory in all prospective trials. Keleidari et al. compared bile reflux frequencies after OAGB and RYGB. They used a Sydney system for classifying histological findings of UGE and self-reported reflux symptoms. They found bile reflux symptoms and a positive bile reflux index in 7.8% and UGE findings suggestive of bile reflux in 4.8% of OAGB patients. These findings were not statistically different compared to the RYGB group, and they concluded that bile reflux is as often encountered after OAGB and RYGB [10]. However, the Sydney system is not intended for postoperative assessment, and it should include biopsies of the antrum, which cannot be obtained after bariatric bypass surgery [11]. Also, the findings of Keleidari et al. are not supported by the findings of the YOMEGA trial, where bile was found in the gastric pouch of 16% of patients after OAGB and none after RYGB [4].

In our study, postoperative UGE findings and BRS findings were significantly associated. Also, reflux symptoms were significantly associated with both BRS and postoperative UGE findings. We found new symptoms in 20% of patients. These symptoms are not specific for bile reflux and can also be due to gastro-esophageal reflux. Similar symptoms have also been reported after a sleeve gastrectomy in 28.1% of patients [12]. According to a recent meta-analysis by Mahawar et al., 0.6–10% of patients had some form of gastro-esophageal reflux symptoms after OAGB [3].

Measuring bile reflux is difficult, and the only non-invasive and accurate method is bile reflux scintigraphy [9]. There are no specific questionnaires to separate symptoms of bile reflux from gastro-esophageal reflux. According to a recent paper by Deitel and Rutledge, all postoperative reflux symptoms after OAGB are easily deemed bile reflux instead of gastro-esophageal reflux without further tests [13]. On the other hand, even significant bile reflux after OAGB can be interpreted as gastro-esophageal reflux. Gastric bile reflux is also to some extent a physiologic phenomenon. Chen et al. studied scintigraphies of unoperated patients with a clinically significant duodenogastric reflux disease (DGR) and healthy controls. Intragastric bile reflux activity was significantly higher in patients suffering from DGR, but healthy controls also had as high as 8% intragastric bile reflux activity in scintigraphies [14].

In light of this evidence, it is fair to say that postoperative bile reflux in the gastric tube after OAGB is a common finding with clinical relevance, and it can be objectively measured. The long-term effects of bile exposure remain to be seen after a longer follow-up. Patients in this study have been enrolled in a randomized controlled study, which is planned to have follow-up for minimum 10 years.

Bariatric societies IFSO and ASMBS have both acknowledged OAGB as an effective bariatric surgery operation; yet, there is continuous doubt about the adverse effects of OAGB [15, 16].

Bile acids have a carcinogenic effect on esophageal mucosa [17, 18]. Potential carcinogenic risk of postoperative bile reflux after OAGB has been proposed numerous times. However, to date, only one known case of esophageal adenocarcinoma after OAGB has been reported [19]. Notably, the patient did not have preoperative endoscopy and the carcinoma was detected already at 1.5 years after OAGB; hence, it is unlikely that the carcinoma developed due to de novo bile reflux after OAGB. Even if there has not been evidence of carcinogenesis after OAGB, bile reflux can cause debilitating symptoms and mandate a reoperation. The recent YOMEGA trial showed serious adverse effects after OAGB, especially malnutrition and also bile reflux. Two patients (1.6%) required conversion to RYGB due to intractable bile reflux [4].

All bariatric surgery techniques have their pros and cons. In this paper, we have not reported the desired outcomes after OAGB, which will be described in detail in future papers regarding this current trial. The decision to undergo bariatric surgery and the chosen technique should always be based on careful consideration of the risks and benefits and the patients should be informed accordingly. Adequate follow-up, including endoscopies, when needed, is of utmost importance.

Our current study has several strengths. It is the first prospective study to objectively study bile reflux with a scintigraphic method and endoscopy before and after OAGB. The follow-up rate is 95%, and all patients were evaluated clinically and endoscopically by a small team of surgeons. The scintigraphic method is a well-documented, non-invasive, and accurate tool for objective assessment of bile reflux.

As weaknesses can be mentioned that the study population is fairly small, and the follow-up time is still short. We did not use a symptom questionnaire because, to our knowledge, there is no questionnaire specifically validated for bile reflux symptoms. The authors interviewed all our patients, and therefore the symptoms are reliably reported. The scintigraphic method can also be criticized, because it only provides a short view of bile flow through the intestine, and we did not perform BRS preoperatively. The amount of bile reflux calculated from the dynamic images was approximate, as the measurements were based on planar images and overlapping intestinal or background activity and signal attenuation may disrupt the analysis. In the future, it would be feasible to calculate the actual tracer activity (in Bq) within the suspected reflux volume on the three-dimensional SPECT-CT images and compare it directly to the administered activity (in Bq).

## Conclusion

Bile reflux in the gastric pouch after OAGB is a common finding in scintigraphy and endoscopy. We found evidence of bile reflux in the gastric pouch in roughly one third of patients, with one case of esophageal bile reflux. Endoscopic findings were mostly in the gastric pouch, and macroscopic lesions were rarely found. All in all, bile reflux in the gastric pouch is clinically relevant and the effects of bile-induced chronic inflammation will be studied in future papers. We propose that all prospective trials on OAGB should include UGE preoperatively, and at 5-year intervals, in order to have data on the actual effects of bile exposure to the gastric pouch and esophagus.
